# Linking Temporal Dominance of Sensations for Primary-Sensory and Multi-Sensory Attributes Using Canonical Correlation Analysis

**DOI:** 10.3390/foods11060781

**Published:** 2022-03-08

**Authors:** Nanako Shimaoka, Shogo Okamoto, Yasuhiro Akiyama, Yoji Yamada

**Affiliations:** 1 Graduate School of Informatics, Nagoya University, Nagoya 464-8601, Japan; shimaoka.nanako@h.mbox.nagoya-u.ac.jp; 2 Department of Computer Sciences, Tokyo Metropolitan University, Hino 191-0065, Japan; 3 Department of Mechanical Systems Engineering, Nagoya University, Nagoya 464-8601, Japan; yasuhiro.akiyama@mae.nagoya-u.ac.jp (Y.A.); yoji.yamada@mae.nagoya-u.ac.jp (Y.Y.)

**Keywords:** sensations, canonical correlation analysis, strawberries, bootstrap resampling, time series analysis

## Abstract

Sensory responses dynamically change while eating foods. Temporal dominance of sensations (TDS) methods record temporal evolution and have attracted attention in the last decade. ISO 13299 recommends that different levels of attributes are investigated in separate TDS trials. However, only a few studies have attempted to link the dynamics of two different levels of sensory attributes. We propose a method to link the concurrent values of dominance proportions for primary- and multi-sensory attributes using canonical correlation analysis. First, panels categorized several attributes into primary- and multi-sensory attributes. Primary-sensory attributes included *sweet*, *sour*, *fruity*, *green*, *watery*, *juicy*, *aromatic*, and *light*. Multi-sensory attributes included *refreshing*, *fresh*, *pleasurable*, *rich/deep*, *ripe*, and *mild*. We applied the TDS methods to strawberries using these two categories of attributes. The obtained canonical correlation model reasonably represented the relationship between the sensations in a reductive manner using five latent variables. The latent variables couple multiple primary- and multi-sensory responses that covary. Hence, the latent variables suggest key components to comprehend food intake experiences. We further compared the model based on the dominance proportions and the time-derivatives of the dominance proportions. We found that the former model was better in terms of the ease of interpreting the canonical variables and the degree to which the canonical variables explain the dominance proportions. Thus, these models help understand and leverage the sensory values of food products.

## 1. Introduction

Sensations dynamically change while eating food. Temporal dominance of sensations (TDS) methods [1,2,3] record the temporal evolution of multiple types of subjectively reported sensations. These methods have attracted considerable attention in the last decade [4,5], in part because they are considered more cost-effective than conventional time-series sensory evaluations such as the time-intensity method [6,7,8].

Understanding the relationships between primary sensations such as flavor, taste, and texture, and higher-level sensations or judgments such as freshness and richness of tastes when eating food products is essential for determining the food values. Relationships between primary sensations and higher-level human responses, including emotions, have been intensively researched using static sensory evaluation methods [9,10,11,12,13,14,15,16]. For example, in [10], primary sensations such as tastes and mouth feels of several vegetables and fruits were linked with higher-level of attributes such as *summery*, *refreshing*, and *fresh-made*. These higher-level attributes are judged based on multi-sensory information, including multiple primary-sensory attributes. However, few studies have linked primary- and multi-sensory data over time. Okada et al. [17] developed a method to establish a model of dynamic causality using temporal data of sensations and emotions related to food intake. They modeled the temporal influences among sensory, emotional, and preferential responses to strawberries using vector auto-regression and Granger causalities. Tachi et al. [18,19] developed a method for establishing state-space models of temporal dominance responses for sensations and emotions and used state variables functioning as memories to show how changes in sensory responses dynamically influence emotional responses. Silva et al. [20] computed the relationship between the temporal changes in liking and the TDS or temporal dominance of emotions (TDE) results. However, this study did not focus on the relationships between sensations and emotions. Galmarini et al. conducted TDS and TDE tasks toward coffee during listening musics [21], and investigated how the sensory and emotional attributes covaried. Their analysis was based on dominance duration and did not aim for the temporal evolution of sensory and emotional responses. Other dynamic analyses for TDS methods, such as those performed by [22,23,24] dealt with only primary sensations and did not connect their results with time-evolving multi-sensory or complex sensory responses. Thus, only a few studies have linked the TDS method results for primary- and multi-sensory attributes. In addition, the studies performed by [17,18,19] contain results that cannot be easily interpreted. Thus far, general methods for linking TDS data for primary sensation and higher-level of sensation or feelings have not been established.

This study linked the TDS results for primary- and multi-sensory attributes using canonical correlation analysis (CCA). This method links two groups of variables using latent variables, which explains the relationships among multiple variables in the two groups. One of the biggest differences between this and earlier studies [17,18] that attempt to link TDS and TDE models is the use of past information in the time-series data. Two earlier models [17,18] were retrospective and modeled the TDS and TDE results at a set time point using past information. In contrast, our model is concurrent and computes the change in an attribute of the TDS task for multi-sensory attributes at a set time point using changes in the attributes of the TDS task for primary-sensory attributes at the same time point. Researchers have discussed which retrospective or concurrent model is most appropriate for analyzing time-series data [25,26]. For example, Chen et al. [25] compared retrospective, concurrent, and unified analysis of neuroimaging data. However, researchers have not yet discussed the possibility of using a concurrent model to link TDS tasks. One of the purposes of this study was to investigate this possibility.

We also investigated whether raw or differential time-series data (i.e., temporal dominance curves) of the TDS method are appropriate for CCA. Temporal dominance curves are the main TDS outputs [2] and express the temporal evolution of the proportions in which individual attributes are dominant in sensations (see Section 2.1). Raw time-series data contain long-term trends, but the samples at different moments are not independent, which may violate the assumptions of CCA or other multivariate analysis techniques. The effect of long-term trends is removed by differentiating raw time-series data. However, the loss of equilibrium information due to differentiation can be a disadvantage. Thus far, previous studies have not compared raw and differential time-series data in terms of TDS data. Hence, we establish two models based on raw (i.e., trend model) or differential time-series data (i.e., differential model). Then, we discuss their semantic validity.

We used the TDS methods to investigate the temporal evolution of the primary- and multi-sensory responses toward strawberries and statistically linked these responses by CCA. As aforementioned, thus far, no studies have attempted to link them by using concurrent models. We subsequently investigated whether the raw or differential time-series data produce a semantically more reasonable model. Strawberries are popular fruits used in earlier sensory studies [17,19,27], which can be compared with our results.

## 2. Method for Modeling

### 2.1. TDS (Temporal Dominance of Sensations) Method

**Figure 1:**
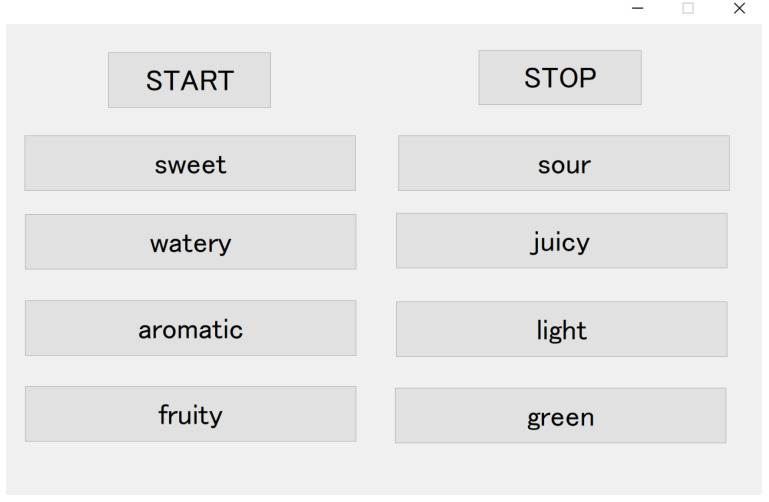
Graphical interface used in a temporal dominance of sensations task. One attribute word is assigned to each button.

The TDS method records the evolution of dominant sensory responses during eating. In this section, we summarize the method. Additional details can be found in [1,4,5]. In the TDS task, a graphical user interface, as shown in Figure 1, is used. Panels push the start button as soon as they put food in their mouth. They then push the button corresponding to the word that best describes the most dominant feelings at each moment. Once the button is pushed, the button continues to be selected until another button is pushed. There is no limit to the number of times a button could be selected. The same button can be selected multiple times, and some buttons may never be selected. When the feelings in the mouth disappear, the panel presses the stop button to end the evaluation. The time when each button is pressed during the task is recorded. This task is repeated a few times for the same food by individual participants.

Individual trials are normalized to analyze the results of the TDS task when the start button is pressed until the stop button is pressed. For a single trial, binary functions that indicate whether the buttons are selected or not at each moment are obtained for each button or attribute. By accumulating the binary functions among all the panels and all trials, the number of times the attribute is selected is compared to the total number of trials. This value is called the dominance proportion and is calculated at each moment. Continuous TDS curves are obtained by smoothing the dominance proportion for each attribute.

### 2.2. Canonical Correlation Analysis (CCA)

**Figure 2:**
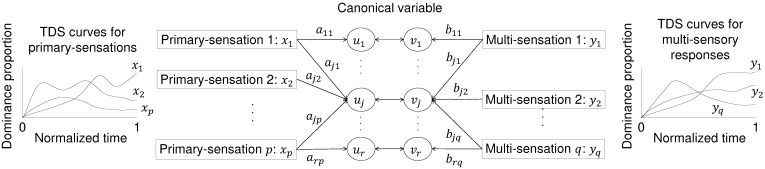
Schematic image of canonical correlation analysis (CCA). CCA connects *p* primary-sensory variables (*x*) and *q* multi-sensory variables (*y*) using the canonical variables *u* and *v*. The coefficients $a_{j}$ and $b_{j}$ were determined to maximize the correlation coefficient between $u_{j}$ and $v_{j}$. j=1,⋯,r (r≤min(p,q)).

CCA is used to estimate the linear relationship between two sets of variables. Figure 2 shows an overview of the model. We computed the relationships between the primary-sensory variables (*x*) and the multi-sensory variables (*y*). These variables indicate the values of the TDS curves at the moment *t*. CCA uses latent variables called canonical variables, of which the number is smaller than or equal to the number of variables included in the smaller set of variables. These values link the variables in the two sets in a reductive manner. When many variables explain primary and multi-sensory sensations, their relationship can be easily interpreted by extracting latent variables.

Suppose that there are *p* primary-sensory attributes and *q* multi-sensory attributes. The zero-centered dominance proportions at instance *t* are denoted by $x_{k}\left(t\right)$ (k=1,⋯,p) and $y_{l}\left(t\right)$ (l=1,⋯,q). We computed the canonical variables $u_{j}$ and $v_{j}$ as

(1) $$\begin{array}{c} u_{j}\left(t\right)=\sum_{k=1}^{p}a_{jk}x_{k}\left(t\right) \end{array}$$

(2) $$\begin{array}{c} v_{j}\left(t\right)=\sum_{l=1}^{q}b_{jl}y_{l}\left(t\right) \end{array}$$

by linear combinations of $x_{k}$ and $y_{k}$ with the coefficients $\left(a_{j1},\cdots \;\;,a_{jp}\right)$ and $\left(b_{j1},\cdots \;\;,b_{jq}\right)$. These coefficients were determined to maximize the correlation coefficient between $u_{j}$ and $v_{j}$. For this calculation, the time functions were discretized into *s* instances, t∈{0,Δt,…,1−Δt,1}. Here, t=0 and t=1 denote the start and end of the normalized task duration, respectively. Further, Δt is the sampling interval: Δt=1/(s−1).

The correlation coefficient between $u_{j}$ and $v_{j}$ is greater than that between $u_{j^{\prime }}$ and $v_{j^{\prime }}$ when $j<j^{\prime }$. Furthermore, $u_{j}$ and $u_{j^{\prime }}$ ($j\neq j^{\prime }$) are independent of each other. This is also true for $v_{j}$ and $v_{j^{\prime }}$. In CCA, the number of canonical variables can be arbitrarily determined, with the maximum being min(p,q). This study determined the number of variables using Pillai-Bartlett’s test.

## 3. Attribute Selection and Categorization

### 3.1. Task

First, we used a questionnaire to select the attributes used in the two types of TDS tasks. Panels ate strawberries and, in a check-all-that-apply manner, selected adjectives that expressed what they felt while eating. The adjective list was prepared by referring to [27,28] and other literature, including websites and food magazines. The list contained 218 adjectives that describe sensations and eating experiences. The adjectives were randomly arranged on a paper and provided to the panels. Then, they categorized the selected attributes into three categories: primary-sensory, multi-sensory, and evaluative. The definition of each category was as follows. Primary-sensory attributes were gustatory, osmatic, and textural aspects. Multi-sensory attributes were related to higher-level sensations that occur while tasting or were based on multiple sensory aspects. Evaluative attributes described the overall significance and importance of the tasting experience and the food value. A similar categorization task was conducted in earlier sensory evaluation tasks (e.g., [29]).

The task was conducted in a quiet office room at approximately 28 °C. The panel ate three strawberries in total before and during the task.

### 3.2. Panels

The panels were 10 university students (8 male and 2 female participants older than 20 years). None of the participants studied or worked in the food industry. They took part in the experiments after providing written informed consent.

### 3.3. Food Specimens: Strawberries

*Tochiotome* strawberries (the cost was approximately 750 JPY for seven strawberries) were used for the experiments. The strawberries were purchased at a fruit market on the day of the experiment. The whole strawberry could be put in the mouth.

### 3.4. Results

Table 1 lists the attributes that gained more than seven votes. The left column in Table 1 lists the attributes that all participants fully agreed upon, with their categories. These attributes include 14 primary-sensory attributes, six multi-sensory attributes, and five evaluative attributes. The primary-sensory attributes were mostly of unisensory cues related to either of taste, osmatic, and texture. One exception may be *green*, which participants agreed to categorize as primary-sensory attributes. *Green* or unripeness is largely judged based on the aroma and taste of unripe and green fruits [27,30]. Although *green* is of multi-sensory cues, the non-food expert participants agreed it as a primary attribute of strawberries. The right column of Table 1 lists seven attributes that spanned multiple categories. Four attributes spanned two categories, and three attributes spanned all the categories. Some attributes such as *earthy* and *caramel* used by experts of strawberries [27] did not gain substantial votes.

To select the attributes for the two types of TDS tasks, we determined the categories of the attributes listed in Table 1 (upper part) by considering the number of votes. In terms of *aromatic*, *rich/deep*, *light*, and *ripe*, we decided their categories based on panel discussion and interviews with the participants. *Aromatic* was categorized as a primary-sensory word because it largely depends on osmatic aspects for strawberries. *Rich/deep*, both of which were semantically similar and were attributed to multiple intensive tastes of strawberries, were categorized as multi-sensory attributes. *Light* was categorized as primary-sensory attributes because several participants mentioned that *light* was related to the duration of sweet taste or weakness of tastes, and *sweet* and *weak* were also categorized as primary-sensory words. *Ripe* was categorized as multi-sensory words since ripeness is judged based on multiple sensory attributes comprising taste and smell. Finally, in terms of the attributes in Table 1, *aromatic* and *light* were categorized as primary-sensory attributes, *rich/deep*, *ripe*, and *mild* were categorized as multi-sensory attributes, and *wonderful* and *elegant* were categorized as evaluative words.

## 4. Experiment: TDS Tasks for Primary- and Multi-Sensory Attributes

### 4.1. Words Used in the Tasks

Informally, eight domestic members in the authors’ institute conducted preliminary TDS tasks using the set of primary- and multi-sensory attributes described in Section 3. In a single task, only one attribute set was used, in line with ISO 13299 [2]. The attributes whose dominance proportions did not reach significance were removed. The significance level was calculated by using the binomial distribution [2]. As a result, eight primary-sensory attributes and six multi-sensory attributes were selected for the main experiments. Table 2 lists the attributes used in the main TDS tasks and their descriptions. We did not use evaluative attributes because there are few earlier studies on temporal dominance tasks for evaluative attributes and the protocol has yet to be established.

### 4.2. Tasks

Before conducting the main tasks, the panels agreed with the descriptions of attributes in Table 2 and used graphical user interfaces to familiarize themselves with the TDS tasks. The attribute buttons were randomly configured on the interfaces for individual panels. The graphical user interface such as the one shown in Figure 1 and data collection software were made by the authors and run on Matlab (ver. 2019a, Mathworks Inc., Natic, MA, USA).

In the main tasks, the participants rinsed their mouths before each trial. They selected a strawberry with their non-dominant hand, keeping their eyes closed and pinching their nose by the dominant hand. Some properties of strawberries including freshness are influenced by their appearances [31]; hence, we shut out the visual cues. Further, their odors could influence the task even before eating; hence, we also shut out the olfactory cues. After they put the whole strawberry into their mouth, they immediately started the evaluation using the graphical user interface with their eyes opened and their hand released from their nose. The panels then selected the button corresponding to the most dominant feeling at each moment, as mentioned in Section 2.1. There was no time limit for the task. Each task lasted approximately 30 s. Each panel performed three trials for each category of sensory attributes. Nine participants performed the task using primary-sensory attributes first, while eight participants performed the task using multi-sensory attributes first. The experiments were conducted at least 1 h after a meal.

### 4.3. Panels

The panels included 17 university students (14 males and 3 females, age: 20–25) from Nagoya University. They were called for by using posters in the university buildings. None of them studied or worked in the food industry and had declared sensory disabilities. They took part in the experiments after providing written informed consent.

### 4.4. Results

**Figure 3:**
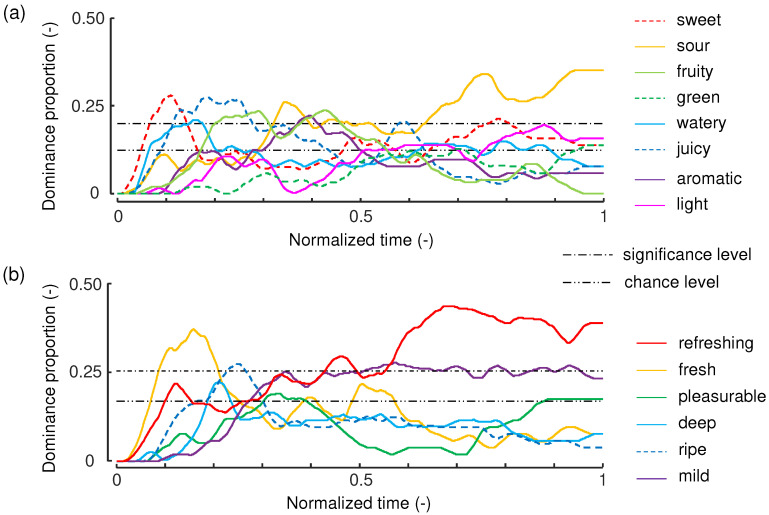
Dominance proportion curves for strawberries. (**a**) TDS curves of primary-sensations. (**b**) TDE curves of multi-sensations.

Figure 3 shows the dominance proportion curves obtained from the tasks. The horizontal axis is the time normalized by the time from the moment the start button was pressed to the time the stop button was pressed (average: ∼30 s). The curves were smoothed using a moving average filter with a time window of 1/30 normalized time.

Regarding primary-sensory attributes, *sweet*, *juicy*, and *watery* were dominant at the beginning of the evaluation (0–0.2 normalized time). Attributes related to fragrance, such as *fruity* and *aromatic*, became dominant at 0.2–0.3 normalized time. Sourness was prominent from the middle to the second half of each task. Similar curves were observed in the results of the TDS method for strawberries [17]. Regarding the multi-sensory attributes, *fresh* was dominant at the beginning. Then, *ripe* and *deep* became prominent. *Refreshing* and *mild* were mostly dominant after approximately 0.3 normalized time. In particular, *refreshing* was prominent at the end of the task.

## 5. Model Linking Primary- and Multi-Sensory Attributes

### 5.1. Data Analysis

We discretized the normalized time into 30 instances, as described by [17]. In our study and [17], a single evaluation task took approximately 30 s on average. It took a minimum of approximately 1 s from when the participant selected a certain button to when they selected the next button. We also obtained the difference between the dominance proportions at time *t* and t−Δt as the differential value at *t*. The first two discretized points were not used in the model computation. This is because almost no descriptors were selected immediately after the start button was pressed in the TDS methods. As mentioned in Section 2.2, we computed CCA for the dominance proportions of sensory attributes using *R* 4.0.3 (cca 1.2.1, ccp 1.1).

### 5.2. Bootstrap Resampling

Using a general data analysis method for TDS methods [2], a single set of dominance proportion curves is obtained from all the panels. As mentioned above, the curves are discretized into 30 instants to compute the CCA. We obtained 28 samples from a single set of curves, excluding the first two discretized instants. However, CCA requires more samples to avoid overfitting. Hence, we increased the number of samples by bootstrap resampling, as described in [32,33,34]. We resampled 51 samples with replacements from the original sample set (17 participants × three repetitions, 51 samples total) obtained in the TDS tasks to calculate a set of TDS curves. We then repeated this process ten times. Subsequently, we obtained ten sets of TDS curves for each primary- and multi-sensory attribute, including 300 and 290 discretized instants for the trend and differential models, respectively. As mentioned above, we did not use the first two instants. Hence, the number of discretized instants used in the analysis were 280 and 270 in the trend and differential models, respectively.

### 5.3. Number of Canonical Variables

Table 3 lists the contribution proportions of the canonical variables for the trend and differential models. According to Pillai-Bartlett tests with a significance level of 0.05, five and four canonical variables were adopted for the trend and differential models, respectively. For the trend model (Table 3 (upper part)), the five canonical variables covered 73% of the variance of the dominance proportions of primary-sensory attributes and 87% of the variance of the multi-sensory attributes. For the differential model (Table 3 (lower part)), four canonical variables captured 50% and 58% of the variance of the differential dominance proportions of primary- and multi-sensory attributes, respectively.

### 5.4. Canonical Correlation Models and Interpretation of Canonical Variables

#### 5.4.1. Models Computed from Trend TDS Curves

**Figure 4:**
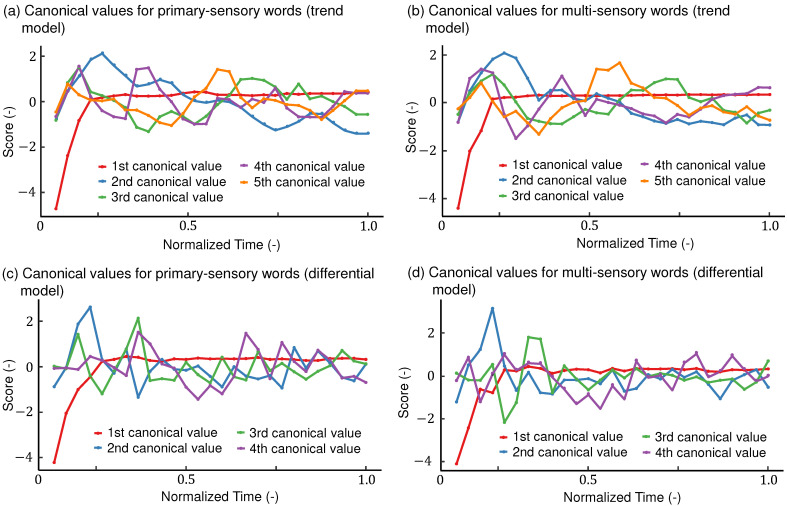
Temporal changes of canonical values corresponding to the primary-sensory (**a**,**c**) and multi-sensory attribute words (**b**,**d**). (**a**,**b**): Canonical values of the trend model. (**c**,**d**): Canonical values of the differential model.

Figure 4a,b show the time series of the canonical values of the trend model. As shown in Table 4, the first canonical values of the primary- and multi-sensory attributes were nearly equally affected by all the attributes. The first canonical variables determined the average size of the TDS curves for all attributes. As shown in Figure 4a,b, the first canonical values were small at the beginning of the task and were almost constant after 0.2 normalized time. As mentioned before, buttons may not be selected immediately after the task starts in the TDS tasks. Thus, the initial values of the dominance proportion curves are small. The first canonical variables show whether a time point is at the beginning of the task or not. It is not important to understand the connection between primary and multi-sensory attributes. A similar latent factor was reported in the principal component analysis of TDS curves [35].

Regarding the second canonical value, *juicy* and *fruity* had a greater influence (Table 4). These values can be interpreted as representing the fruitiness of taste and smell. In the multi-sensory attributes, *ripe* was prominent. The correlation coefficient of the second canonical variables between the primary- and multi-sensory attributes was as large as 0.83 (Table 4). According to Table 3, the contributions of the second canonical variables were the largest among all the canonical variables for the primary- and multi-sensory categories. The values of these canonical variables were large in the first half of the task, peaked around 0.25 normalized time, and then gradually decreased. A study using principal component analysis of static sensory evaluation of strawberries [27] reported that the fruity aroma and juiciness of strawberries are the most prominent features for classifying ripe strawberries. The features in [27] correspond to the second canonical variables in the present study.

For the third canonical variable, as in Table 4, *watery*, has the highest coefficients in the sensory attributes, followed by *light* and *green*. *Refreshing* is representative of the multi-sensory attributes. Hence, this canonical value can be interpreted as a cool feeling (*light*, *green*, and *refreshing*) with high water content. The correlation between the third canonical variables for the primary- and multi-sensory attributes was 0.47 (Table 4). The contribution to the primary-sensory attributes was 0.10, and that to the multi-sensory attributes was 0.16 (Table 3). The third canonical values are high at 0.1–0.2 normalized time, indicating that the water content in the strawberries was felt after the first bite (Figure 4a). The values were also high near and after 0.6–0.7 normalized time, when *refreshing* is prominent (Figure 4b).

Regarding the fourth canonical variables, *juicy*, *sour*, and *watery* exhibited large coefficients among the sensory attributes. These results are consistent with those of Oliver et al., where juiciness and sourness constituted a major component of the static sensory analysis for strawberries [30], although they did not use watery attributes in their study. *Fresh* exhibited a large coefficient among the multi-sensory attributes, followed by *pleasurable* and *refreshing*. The correlation between the canonical variables was moderate (0.36), and the contribution rates were 0.07 and 0.08 for the sensory and multi-sensory attributes, respectively, which were the smallest among all the canonical variables. These results suggest that the fourth canonical variables may not be important in explaining the behavior of the entire dominance proportion curves. The fourth canonical variables are interpreted as features of strawberries, with a lot of pulp, juice, and sourness, leading to perceived freshness.

For the fifth canonical variables, *green*, *juicy*, and *sweet* were prominent among the primary-sensory attributes. Among the multi-sensory attributes, *fresh* and *mild* were prominent. The fifth canonical variables exhibited peaks at the beginning and middle of the task, as shown in Figure 4a,b. These canonical values are difficult to interpret. According to [27,30], sweet and green flavors of strawberries are perceptually opposite, which does not match the coefficients of the fifth canonical variables. The correlation coefficient of the fifth canonical variables between the primary- and multi-sensory attributes was low (0.25), and the contribution rates were 0.09 and 0.12 for the primary- and multi-sensory attributes, respectively. Therefore, the fifth canonical variable may not be important for interpreting the relationships among the TDS curves of dominance proportions of strawberries.

#### 5.4.2. Models Computed from Differential TDS Curves

Figure 4c,d show the time series of canonical variables when the differential curves of dominance proportions were used for CCA. The contribution ratios of all the canonical variables were similar, ranging from 0.10 to 0.21 (Table 3). The first–fourth canonical variables explained 50% and 58% of the differential curves of temporal dominance for primary- and multi-sensory attributes, respectively. For the multi-sensory attributes, the contribution ratios of the fifth and sixth canonical variables that were rejected by the Pillai-Bartlett test were relatively large, suggesting that the model could not effectively summarize the differential curves of the dominance proportions.

As listed in Table 5, the first canonical variables were equally affected by all attributes in the primary- and multi-sensory categories. These variables indicate the trends common to all the attributes. As shown in Figure 4c,d, the canonical variables were small at the beginning of the task and were generally constant after 0.2 normalized time. Similar to the first canonical variables for the trend model in Section 5.4.1, the first canonical variables for the differential model indicate the elapsed time and do not contribute to understanding the relationship between the primary- and multi-sensory attributes.

As shown in Table 5, the second canonical variables are positively affected by *light* and *fruity* and negatively affected by *sweet* and *watery*. *Rich/deep* influenced the canonical variable among the multi-sensory attributes. Overall, these results are difficult to interpret. It seems semantically reasonable that *rich/deep*, which indicates strong taste, increased as *fruity* increased and *watery* decreased. However, it seems paradoxical that strawberries feel richer and deeper when lighter and less sweet. These canonical values exhibited a peak around 0.2 normalized time and continued to have a value close to 0 after that. The correlation coefficient between the two canonical variables was 0.55, and the contribution ratios were 0.17 and 0.16 for the primary- and multi-sensory attributes, respectively.

For the third canonical variables describing the primary-sensory attributes, *fruity* negatively affected the canonical variable, and *aromatic* and *sour* positively affected the variable. Among the multi-sensory words, the negative influence of *ripe* was the most prominent, followed by positive influences by *rich/deep* and *refreshing*. Some of these connections were easy to interpret, whereas others were difficult to interpret. In a sensory study of strawberries [27], strawberries with a stronger fruity aroma and less sourness were judged to be riper, which is partly in line with the third canonical variable. In contrast, *fruity* (defined as the aroma of sweet fruits) and *aromatic* (pleasant smell) had opposite directions. Further, *ripe* and *rich/deep* also had opposite directions. The opposite directions for *fruity*, *aromatic*, *ripe*, and *rich/deep* appear contradictory, which makes the interpretation of the third canonical variables less intuitive. The correlation coefficient of the third canonical variables between the primary- and multi-sensory attributes was as small as 0.29.

Regarding the fourth canonical variables, *green* and *light* exhibited a strong influence on the canonical variable among the primary-sensory attributes. Furthermore, *pleasurable* was the most influential multi-sensory attribute. As shown in Figure 4c,d, these canonical variables were roughly neutral or positive at the beginning and end of the task and negative at the middle of the task. However, the values fluctuated rapidly, and no clear trends were observed. The correlation coefficient between the canonical values for sensory and multi-sensory attributes was 0.26. The meaning of the fourth canonical variables can be easily interpreted. Green-smelling and light-tasting strawberries lead to negative (unpleasurable) feelings.

## 6. Discussion

Our study results show that trend or differential models are suitable for computing the CCA of the concurrent values in dominance proportion curves. In the trend model, five pairs of canonical variables connected primary- and multi-sensory attributes. According to Table 3, 73% and 87% of the variances of the dominance proportion values of the primary- and multi-sensory attributes, respectively, were explained by these canonical variables. These values may be compared with those of trajectory plots of TDS curves in earlier studies. For example, Nguyen et al. [35], Lenfant et al. [36], Merlo et al. [37], and Nguyen and Wismer [38] reported that principal components explained 52–83% of the variances of temporal dominance curves while eating wheat flakes, yoghurts, and hamburgers, respectively. Although there is no standard about these values, those in the present study and earlier studies are comparable, indicating that the TDS curves were well explained by the latent variables. The number of latent variables was smaller than the number of multi-sensory attributes (six) by only one variable. Thus, the contraction effect of the model was not large. The meaning of four of the five canonical variables can be reasonably interpreted, but it is difficult to interpret the fifth canonical variables. In the differential model, primary- and multi-sensory attributes were connected by four pairs of canonical variables. According to Table 3, 50% and 59% of the variances in the differential dominance proportions of primary- and multi-sensory attributes, respectively, were explained. Interpreting the second and third canonical variables was difficult. Considering these points comprehensively, the trend model is more suitable for analyzing dominance proportion curves than the differential model. However, we cannot conclude that either model is superior solely from the examples of strawberries. Similar studies are necessary for other foods in the future.

**Figure 5:**
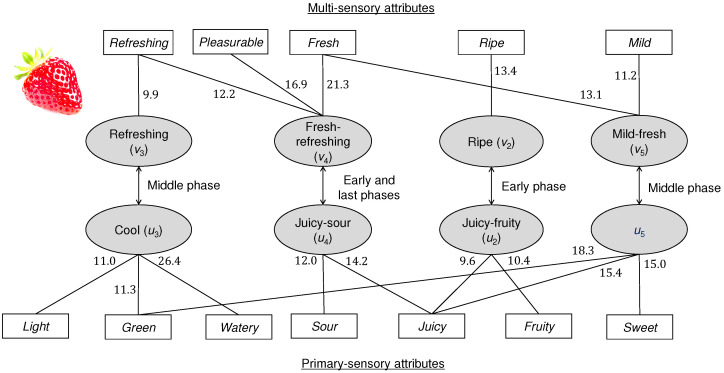
Trend model for strawberries. Four pairs of canonical variables are shown, except for the first canonical variables. The canonical variables were named by considering the prominent coefficients of the attributes in Table 4. The fifth canonical variable ($u_{5}$) for the primary-sensory attributes is not named. The major connections are shown. Values nearby the lines indicate their coefficients.

Figure 5 summarizes the trend model for strawberries with major connection lines between the attributes and canonical variables. Note that the first canonical variable pair indicates the time elapsed and is not shown. The canonical variables were named for convenience. These four types of canonical variable pairs determine the dynamic behavior of the dominance proportions of strawberries. In the early phase of the eating experience, juicy-fruity (second canonical variable) and juicy-sour (fourth canonical variable) factors are prominent primary-sensory aspects. These factors are likely caused by the juice coming out after initial biting and indicate that the fruity aroma and taste associated with the juice caught the attention of the panels. The participants also felt the ripeness and freshness of strawberries during the early phase. This agrees with [31] where the freshness of strawberries was largely judged by juiciness after their visual factors such as the surface shine and bruises. In our model, ripeness was not related to *green* whereas unripe strawberries were characterized by the green taste and aroma in a study using strawberries [27]. This difference may be because underripe strawberries were intentionally included in [27] whereas apparently underripe strawberries were not used in the present study. The third and fifth canonical variables were relatively large in the middle phase. As shown in Figure 3a, no sensory attributes were dominant in this phase, leading to a feeling of mildness (represented by the fifth canonical variable pair). Subsequently, a refreshing feeling becomes dominant (as represented by the third canonical variable). The last phase is represented by the fourth canonical variable pair, which represents an intensely sour taste and refreshing feeling. *Refreshing* was defined as “pleasantly cool” as in Table 2. In general, this attribute is favorable in evaluating fruits [39]. In [39], *refreshing* was felt for juicy, sweet, and sour mandarins, and *refreshing* appeared to be judged based on multiple types of basic tastes. For the strawberries in the present study, *refreshing* was connected with *sour* and *juicy* by the fourth canonical variable. Furthermore, *refreshing* was prominently connected with *watery* by the third canonical variable. The meanings of *refreshing* may differ among different types of fruits; however, regarding strawberries and mandarins, sourness is a potentially common factor. For strawberries, sourness in the last eating phase is generally accepted by consumers [40].

Our study suggests that the concurrent model can link the results of the TDS methods for primary- and multi-sensory attributes. However, in order to determine which of the concurrent or retrospective models are more suitable, a variety type of foods need to be tested whereas only strawberries were tested in the present study. The two models, i.e., the concurrent model in this study and retrospective models in [17,18] contain some points that remain semantically validated. Both models need further studies.

We conducted two types of TDS tasks by using primary- and multi-sensory attributes. According to ISO standards [2], very different attributes, such as sensory and emotional attributes, should not be tested in the same temporal dominance tasks. However, there is no general method to classify very different sensory attributes. Hence, following earlier studies [29], we classified attributes into three categories as in Section 3. We found that attribute categorization is difficult because some attributes span multiple categories. A similar problem was reported in [29] where several attributes among more than one hundred for describing experiences related to touch were categorized as both sensory and evaluative. The panels in the present study categorized *pleasurable*, which is the translation of *kokochiyoi* in Japanese, into multi-sensory; however, *pleasurable* also may include the aspect of evaluative attributes. *Kokochiyoi* can be also translated as comfortable and delightful. The results of attribute categorization task may depend on the cultures and expertise of the panels considering that some attributes used by the experts, such as *earthy* and *caramel* [27] were not selected by the non-expert panels in the present study. Different categorization leads to different results of CCA, which limits the generality of our approach. Furthermore, the present study lacks a demonstration of uncertainties of canonical variables. As shown in Figure 4, some canonical values fluctuate around zero at some moments. It is meaningful to know whether those values are statistically different from zero.

## 7. Conclusions

Despite the growing usage of TDS methods in food industries, no previous studies have proposed to link the TDS curves for the sensory attributes in different categories. We established a model to link these curves using the concurrent dominance proportions and CCA values. We investigated whether the trend or differential models are better in terms of the semantic validity of the canonical variables and the dominance proportions explained by the canonical variables. We then determined that the trend model is better for strawberries, in which five pairs of canonical variables represent the temporal evolution of the dominance proportions. The proposed method will increase the comprehension of the dynamic relationships between primary- and multi-sensory attributes while eating foods based on fewer latent factors, i.e., canonical variables. The method is expected to be useful especially when the number of attributes in the two categories is large. We also found that categorizing primary- and multi-sensory attributes are not easy, and even the trend model includes a semantically unclear pair of canonical variables.

## Figures and Tables

**Table 1:** Results of the attribute selection task. The attributes gained more than 7 of the 10 votes. (upper part) Attributes that the panels fully agreed upon and their categories. (lower part) The panel’ opinions on categorization disagreed. The values in parentheses are the number of votes for each attribute.

Primary-Sensory	Multi-Sensory	Evaluative
sweet	refreshing	delicious
sour	pleasurable	satisfied
hard	unripe	good
soft	fresh	luxury
watery	loving	
crispy		
juicy		
moist		
green		
fruity		
berry		
weak		
strong		
smooth		
**Primary-Sensory**	**Multi-Sensory**	**Evaluative**
aromatic (3)	aromatic (4)	
rich/deep (4)	rich/deep (4)	
	wonderful (3)	wonderful (5)
	elegant (3)	elegant (4)
light (4)	light (3)	light (2)
ripe (3)	ripe (3)	ripe (4)
mild (2)	mild (3)	mild (2)

**Table 2:** Attributes used in the TDS tasks.

Primary-Sensory Attributes	Description
Sweet	Basic taste. No definition was provided.
Sour	Basic taste. No definition was provided.
Fruity	Smell of sweet fruits.
Green	Smell, taste, and mouth feel of grass or unripe fruits.
Watery	Water content with no strong taste.
Juicy	Amount of juice and flesh.
Aromatic	Complex but pleasant smell.
Light	Sweet taste that does not last long in the mouth.
Multi-sensory attributes	
Refreshing	Pleasantly cool.
Fresh	Recently harvested.
Pleasurable	Feeling of pleasure.
Rich/deep	Combination of multiple strong tastes or aromas.
Ripe	Fully grown and ready to be eaten.
Mild	Taste spreads gently without strong stimuli.

**Table 3:** Contribution of canonical variables to the sample variances in the (top) trend model and (bottom) differential model. The canonical variables are arranged in the order of correlation coefficients between the dominance proportions of primary- and multi-sensory attributes.

Trend Model					
Canonical	Contribution to	Contribution to	Pillai-Bartlett	*F*-Value	*p*-Value
Variable	Primary-Sensations	Multi-Sensations	Trace		
1st	0.14	0.20	2.06	17.7	0.000
2nd	0.33	0.31	1.12	10.7	$3.2\times 10^{-14}$
3rd	0.10	0.16	0.42	5.20	$3.3\times 10^{-14}$
4th	0.07	0.08	0.21	3.93	$5.2\times 10^{-7}$
5th	0.09	0.12	0.08	2.69	0.006
6th	0.09	0.13	0.01	1.17	0.319
**Differential Model**					
Canonical	Contribution to	Contribution to	Pillai-Bartlett	*F*-Value	*p*-Value
Variable	Primary-Sensations	Multi-Sensations	Trace		
1st	0.05	0.10	1.38	10.1	0.000
2nd	0.17	0.16	0.48	4.03	$4.0\times 10^{-14}$
3rd	0.14	0.15	0.18	2.08	0.002
4th	0.14	0.17	0.09	1.71	0.044
5th	0.13	0.21	0.02	0.85	0.556
6th	0.13	0.20	0.01	0.47	0.703

**Table 4:** Coefficients for 1st–5th canonical variables when the raw (i.e., trend) TDS curves were adopted for computing CCA. Correlation coefficients were computed between the two canonical variables for the primary- and multi-sensory attributes for each of the five canonical variables.

Canonical Variables					
Primary-sensory	1st	2nd	3rd	4th	5th
sweet	17.4	5.5	5.0	0.1	15.0
sour	17.1	2.6	3.8	12.0	3.5
fruity	17.6	10.4	4.8	−2.8	3.0
green	17.5	1.0	11.3	2.9	18.3
watery	15.7	5.8	26.4	9.8	2.9
juicy	16.4	9.6	5.9	14.2	15.4
aromatic	17.0	4.0	8.6	6.8	6.3
light	17.8	4.7	11.0	5.1	3.3
Correlation	0.97	0.83	0.47	0.36	0.25
Multi-sensory	1st	2nd	3rd	4th	5th
refreshing	11.8	0.8	9.9	12.2	4.7
fresh	11.5	7.2	4.5	21.3	13.1
pleasurable	11.8	6.2	1.4	16.9	−5.1
rich/deep	12.0	5.2	4.1	9.5	−1.5
ripe	11.4	13.4	6.9	−2.5	4.9
mild	12.0	3.2	4.4	5.4	11.2

**Table 5:** Coefficients for 1st–4th canonical variables when the differential values of the dominance proportion curves were used for CCA.

	Canonical Variables			
Primary-Sensory	1st	2nd	3rd	4th
sweet	18.9	−7.2	−6.5	1.5
sour	16.1	3.2	9.7	1.9
fruity	18.5	12.0	−13.4	8.0
green	17.0	−0.87	−5.9	−29.9
watery	16.9	−9.3	−7.4	−3.6
juicy	18.1	−2.5	5.9	−5.5
aromatic	18.1	1.1	13.4	2.2
light	16.6	17.6	6.6	−15.2
Correlation	0.95	0.55	0.29	0.26
Multi-sensory	1st	2nd	3rd	4th
refreshing	14.0	−7.5	10.0	−3.2
fresh	15.0	−3.9	7.2	−5.2
pleasurable	13.2	2.2	4.1	26.6
rich/deep	11.2	21.0	12.9	−0.79
ripe	14.1	7.1	−21.0	−0.83
mild	13.2	8.4	4.5	−7.4

## Data Availability

The data used in the present study are available at TDbear project at https://www.comp.sd.tmu.ac.jp/hci/tdbear/ (accessed on 5 March 2022).

## Abbreviations

The following abbreviations are used in this manuscript:

TDS	Temporal dominance of sensations
CCA	Canonical correlation analysis
